# The TCRAT Technique (Total Coronary Revascularization via Left Anterior Thoracotomy): Renaissance in Minimally Invasive On-Pump Multivessel Coronary Artery Bypass Grafting?

**DOI:** 10.3390/jcdd13010028

**Published:** 2026-01-04

**Authors:** Volodymyr Demianenko, Hilmar Dörge, Christian Sellin

**Affiliations:** Department of Cardiothoracic Surgery, Heart-Thorax Center, Klinikum Fulda, University Medicine Marburg, Campus Fulda, Pacelliallee 4, 36043 Fulda, Germany; demcardio@gmail.com (V.D.); christian.sellin@klinikum-fulda.de (C.S.)

**Keywords:** TCRAT, minimally invasive coronary surgery, CABG, multiarterial coronary revascularization

## Abstract

Total Coronary Revascularization via left Anterior Thoracotomy (TCRAT) represents a modern evolution of sternum-sparing, on-pump multivessel coronary artery bypass grafting. In this review, we will summarize the historical development, detail the surgical principles, and provide a comprehensive overview of the clinical outcomes of TCRAT. The technique combines cardiopulmonary bypass using peripheral arterial as well as venous cannulation and cardioplegic cardiac arrest using transthoracic aortic cross-clamping with surgical access through a left anterior minithoracotomy. By applying special slinging and rotational maneuvers, both a stable exposition of all coronary territories—in particular those of the right and the circumflex coronary artery—and a quiet, bloodless operating field enable complete anatomical revascularization and complex coronary surgery procedures, including all variations in multiarterial grafting in unselected patients. Data from all published clinical series were integrated, and a weighted analysis of a total of 2282 patients was performed. TCRAT proved to be very effective with regard to complete anatomical revascularization and modern grafting strategies, and it showed excellent perioperative safety in an all-comers population. Both the 30-day mortality and perioperative stroke incidence were distinctly below 1.0%. Data from mid-term follow-up, although rare so far, are promising and compare well to those of the important RCTs. The TCRAT approach eliminates sternal complications completely and accelerates recovery. As an on-pump arrested-heart surgery, TCRAT inherently permits the combination of minimally invasive multivessel CABG with a variety of other cardiac operations, mainly the combination with valve procedures. The integration of robotic and endoscopic assistance represents the next evolutionary step. With its reproducibility and broad applicability, TCRAT holds strong potential to become a standard routine technique in the field of minimally invasive cardiac surgery.

## 1. Introduction

Since its introduction in the early 1960s, coronary artery bypass grafting (CABG) has evolved into one of the most frequently performed and extensively studied surgical treatments for coronary artery disease. For decades, the conventional full-median sternotomy approach has been regarded as the standard of care, providing excellent exposure and reproducibility. However, it remains a highly invasive procedure associated with significant surgical trauma, postoperative pain, delayed functional recovery, and the risk of sternal wound complications, particularly in obese and diabetic patients. These limitations have driven the continuous pursuit of less invasive alternatives over the past three decades.

Beginning in the mid-1990s, numerous innovative strategies were introduced to reduce the invasiveness of CABG. Both on-pump and off-pump concepts were explored, with procedural refinements aimed at minimizing incisions, preserving sternal integrity, and improving postoperative outcomes. The evolution included video-assisted, robotic-assisted, and totally endoscopic approaches, often in combination with smaller thoracic incisions. Despite these advances, the term “minimally invasive CABG” still lacks a universally accepted definition, as the degree of invasiveness varies considerably among techniques and centers.

This review focuses specifically on minithoracotomy-based CABG approaches, tracing the historical progression of on-pump, arrested-heart techniques performed through limited thoracic access. It highlights the conceptual and technical evolution leading to the development of Total Coronary Revascularization via left Anterior Thoracotomy (TCRAT)—a modern, reproducible method that combines the physiological advantages of conventional on-pump surgery with the benefits of sternum-sparing minimally invasive access.

In the early 1990s, the Stanford group initiated a comprehensive program to develop a platform for performing cardiac surgery through non-median sternotomy incisions, utilizing percutaneous cardiopulmonary bypass (CPB) on an arrested heart. Through extensive experimental work on animal and cadaveric models, the team designed a sophisticated system that included femoral arterial and venous access for CPB, a transfemoral endoaortic occlusion balloon catheter integrating lines for antegrade cardioplegia delivery and aortic root venting, as well as an endopulmonary vent [1]. This “Port-Access” system aimed to replicate all essential components of conventional on-pump surgery while entirely avoiding sternotomy. The first clinical application of the technique, reported in 1997 in a small cohort of 12 patients undergoing CABG through a limited thoracotomy, demonstrated the technical feasibility of performing closed-chest coronary revascularization under full CPB and cardioplegic cardiac arrest [2]. Soon after, several centers worldwide adopted the concept, leading to the establishment of the “Port-Access International Registry,” which by 1999 had accumulated more than 900 isolated CABG cases from 121 institutions, showing acceptable early outcomes [3]. However, the registry was later criticized for underrepresenting the overall learning curve and early complications. Major limitations of the Port-Access approach included the risks of aortic dissection and stroke related to retrograde femoral perfusion, the procedural complexity associated with endoaortic balloon occlusion, and the substantially increased cost and duration of surgery [4,5,6]. Consequently, enthusiasm for the Port-Access system declined in the early 2000s, coinciding with the rise of minimally invasive direct CABG (MIDCAB), LAST (left anterior small thoracotomy) and off-pump “beating-heart” strategies, which had originally emerged in the 1990s.

Despite these challenges, the Port-Access experience provided critical proof-of-concept that multivessel CABG could be performed safely under CPB and cardioplegic arrest via a small lateral minithoracotomy. Moreover, it revealed distinct physiological and clinical benefits compared with conventional sternotomy CABG, including less postoperative pain, a reduced systemic inflammatory and stress response, improved pulmonary function, and faster recovery with higher quality-of-life scores in the early postoperative phase [7,8]. These pioneering developments laid the experimental and conceptual foundation for subsequent generations of sternum-sparing, on-pump minimally invasive coronary revascularization techniques.

To avoid sternotomy and its associated complications while overcoming the limitations of endoaortic balloon occlusion and retrograde perfusion, the Dresden group developed an alternative strategy for multivessel revascularization under cardiopulmonary bypass [9,10]. Their technique employed a small left anterior thoracotomy placed in the third intercostal space, with the cartilages of the third and fourth ribs partially detached from the sternum to facilitate exposure. Through this access, the left internal thoracic artery (LITA) could be safely harvested under direct vision. Venous cannulation was achieved via the right femoral vein, while the ascending aorta was centrally cannulated through the thoracic incision. A conventional aortic cross-clamp was applied directly under vision, and antegrade cold blood cardioplegia was administered through a small aortic root cannula that subsequently served as a vent. Once decompressed, the relaxed heart could be gently mobilized and rotated, allowing exposure of all major coronary territories, including those of the right coronary and circumflex systems. In this way, complete anatomical revascularization could be achieved through a single limited incision.

The early experience in 102 patients operated on between 1996 and 1998 demonstrated favorable results, with no early mortality, no strokes, and a remarkably low incidence of major complications or conversion to sternotomy. Notably, access to all coronary targets was feasible, and even technically demanding procedures such as bilateral internal thoracic artery use and coronary ostioplasty were successfully performed. In a prospective randomized comparison between the “Dresden technique” and conventional on-pump and off-pump CABG, recovery parameters were comparable, while postoperative pain, mobility, and overall convalescence were significantly improved in the thoracotomy group, despite longer operative times [11].

A related modification, described as the “West Coast technique,” utilized a slightly more lateral anterior thoracotomy through the fourth intercostal space [12]. In this variant, the arterial cannula was introduced through a separate stab incision in the second intercostal space to access the ascending aorta, while venous drainage was again obtained via the right femoral vein. A transthoracic aortic clamp permitted direct cross-clamping, and antegrade cardioplegia was administered through a dedicated aortic root cannula, which was later used for venting. All distal and proximal anastomoses were performed using conventional instruments under a single cross-clamp period. In a cohort of 32 carefully selected patients, this approach yielded encouraging early outcomes, reinforcing the concept that multivessel coronary bypass under full CPB and cardioplegic arrest could be accomplished safely through a limited anterior thoracotomy.

Despite these promising results, both the Dresden and West Coast techniques remained confined to a few specialized centers. The limited adoption likely reflected the high technical demands, prolonged operative times, and lack of dedicated instruments for working through small incisions. Nevertheless, these pioneering approaches provided unequivocal evidence that full multivessel on-pump CABG with cardioplegic arrest could be performed safely and effectively via a small thoracotomy, establishing a crucial conceptual bridge between the early port-access systems and the later development of standardized, reproducible methods such as TCRAT.

Nearly two decades later, a Kyiv group revitalized the concept of minimally invasive CABG for multivessel disease. Their technique utilized a limited left anterior thoracotomy, peripheral cardiopulmonary bypass, and cardioplegic arrest. Building upon earlier principles, the team incorporated a transthoracic clamp and specific rotational maneuvers with vascular slings, thereby enhancing procedural standardization and reproducibility. The initial series of 170 unselected consecutive patients demonstrated excellent early outcomes, confirming the feasibility and safety of this method, later termed Total Coronary Revascularization via left Anterior Thoracotomy [13]. Subsequently, several centers adopted and further refined the TCRAT approach, supporting its ongoing development and clinical validation [14,15,16,17,18,19].

## 2. TCRAT—Surgical Technique

The TCRAT procedure is defined by three principal technical elements [20]. First, the left minithoracotomy is placed as anteriorly as possible to minimize the distance to both the aorta and coronary targets. Second, peripheral CPB cannulation combined with transthoracic aortic cross-clamping provides optimal exposure and working space on an empty, arrested heart. Third, strategic slinging maneuvers around the great vessels enable controlled rotation and elevation of the heart toward the incision, ensuring stable visualization of all coronary territories.

Step 1—Left Anterior Minithoracotomy and ITA Harvesting (Video S1). Demonstration of patient positioning, anterior thoracotomy at the fourth intercostal space, and LITA harvesting technique under direct vision with an asymmetrical retractor.

Patients are positioned supine with an inflatable pad placed beneath the left hemithorax to facilitate internal thoracic artery harvesting and cardiac exposure. The chest is entered through an anterior muscle-sparing thoracotomy, approximately 6–8 cm in length, performed along the fourth intercostal space. After insertion of a small thoracotomy retractor, the intercostal muscles are divided laterally to minimize the risk of rib fracture. Compared with the more lateral incision commonly employed in MIDCAB procedures, this anteriorly positioned approach significantly shortens the distance to both the ascending aorta and the coronary targets, thereby improving reach, visualization, and ergonomics during anastomotic construction.

Dissection is continued medially until LITA is clearly identified. Following the original description of the technique [13], the LITA is divided at the level of the fourth ICS to prevent undue traction on the vessel. An asymmetrical ITA retractor is then positioned, and the LITA is harvested proximally beyond the origin of the left mammary vein under direct vision using long conventional surgical instruments. Single-lung ventilation is optional but generally not required, as adequate exposure can typically be achieved with double-lung ventilation in most patients.

To obtain additional conduit length, a modified two-stage harvesting technique has been introduced [14]. After sufficient mobilization of the LITA at the level of the thoracotomy, the retractor is first positioned caudally, allowing distal harvesting beyond the bifurcation without vessel tension. The retractor is then repositioned cranially to facilitate proximal dissection toward the subclavian artery. This maneuver yields a longer and more mobile graft, expanding the reach to distal coronary targets and enabling more versatile graft configurations, including sequential or composite anastomoses.

Despite these refinements, LITA harvesting through an anteriorly located incision may remain technically demanding due to the steeper angle of approach compared with an anterolateral thoracotomy. In challenging cases, initiation of CPB with subsequent cardiac decompression can improve exposure and facilitate safe dissection. Although harvesting of the right internal thoracic artery (RITA) through the same incision is technically feasible, it remains considerably more demanding and is currently reserved for highly experienced teams. Future refinements are likely to include robotic or endoscopic assistance to further enhance visualization, ergonomics, and safety during ITA harvesting.

Step 2—Peripheral Cannulation, Cardiopulmonary Bypass, and Transthoracic Aortic Cross-Clamping (Video S2). Step-by-step presentation of peripheral cannulation strategy, pericardial opening, antegrade cardioplegia administration, and transthoracic cross-clamping for complete cardiac decompression and exposure.

Peripheral cannulation and transthoracic aortic cross-clamping provide unrestricted direct visualization through the limited anterior thoracic incision. Arterial cannulation for CPB is guided by preoperative computed tomography imaging and is performed via the femoral or right axillary artery, depending on vessel anatomy and aortic calcification. Venous drainage is achieved percutaneously through the femoral vein; in patients with a body surface area exceeding 2.0 m^2^, the addition of a percutaneous jugular venous cannula may be considered to optimize venous return. Vacuum-assisted venous drainage is routinely employed to ensure complete cardiac decompression and maintain a stable, bloodless operative field.

The pericardium is opened longitudinally from the apex toward the ascending aorta and suspended with multiple stay sutures to expand the operative window. The ascending aorta is then mobilized by blunt dissection from the pulmonary trunk and encircled with a traction tape. Gentle traction on this tape brings the aorta closer to the thoracotomy, allowing fingertip access for insertion of a small aortic root cannula. This cannula is initially used for antegrade cardioplegia delivery and subsequently serves as an aortic vent to facilitate continuous decompression during cardiac arrest.

A transthoracic aortic clamp is introduced through a separate small incision in the second intercostal space along the anterior axillary line. After a brief reduction in CPB flow to lower arterial pressure, the clamp is applied under direct vision while gently pulling the aortic traction tape. Antegrade cardioplegia is administered through the aortic root cannula to achieve complete cardiac arrest. Once the heart is motionless and decompressed, the pericardial cavity provides ample working space with excellent hemodynamic stability.

Complete decompression of the heart is of paramount importance, as it permits controlled rotation and elevation of the arrested heart toward the thoracotomy, thereby enabling precise exposure of all coronary territories, including the right and circumflex distributions. This technical principle (creating a stable, fully decompressed operative field) forms the foundation for the subsequent exposure maneuvers and safe construction of distal anastomoses under direct vision.

Step 3—Exposure Maneuvers (Video S3). Illustration of slinging techniques using tapes around the pulmonary veins and inferior vena cava to achieve stable exposure of all coronary territories for standard manual graft anastomoses.

Stable and reproducible exposure of distal coronary targets in TCRAT is achieved through a series of precisely controlled slinging maneuvers that enable full access to all left ventricular territories under direct vision [13,14]. Once the heart is arrested and completely decompressed, separate tapes are placed around the left pulmonary veins (LPV) and the inferior vena cava (IVC). These tapes serve as dynamic traction tools for sequential manipulation of the heart.

For exposure of the inferior wall coronary arteries, the LPV tape is gently passed beneath the heart toward the diaphragmatic pericardium and pulled caudally. This maneuver elevates the apex and rotates the inferior wall upward toward the thoracotomy, effectively reducing the working distance to the posterior surface. Simultaneous upward traction on the IVC tape further shortens the depth of the operative field and stabilizes the heart in a favorable position.

Exposure of the lateral wall is achieved by directing the LPV tape leftward and applying gentle upward retraction on the IVC tape. The left anterior descending artery is accessed by cranial traction on the LPV tape while returning the IVC tape toward the right. The combination of these controlled traction vectors produces a stable, predictable orientation of the heart, minimizing torsion and ensuring optimal visibility of all coronary territories, including the right coronary and circumflex systems.

This system of traction and countertraction effectively reduces the distance from the skin incision to the coronary arteries, allowing safe and ergonomic performance of all distal anastomoses under direct vision. The exposure remains stable even during complex coronary preparation or multiarterial grafting, permitting the use of conventional surgical instruments and standard manual knot-tying techniques without the need for specialized devices or stabilization systems. The reproducibility and simplicity of these exposure maneuvers are key elements of the TCRAT technique, ensuring consistent access to all coronary targets irrespective of anatomical variability or graft configuration.

## 3. TCRAT—Clinical Outcomes

Although this article is intended as a technical narrative review rather than a formal meta-analysis, we sought to enhance transparency and reduce selection bias in the summary of contemporary TCRAT outcomes by applying a PRISMA-informed study identification workflow. MEDLINE/PubMed was searched (last search: 1 September 2025) using relevant MeSH terms (including “Coronary Artery Bypass”, “Thoracotomy”, and “Minimally Invasive Surgical Procedures”) combined with free-text keywords (e.g., CABG, left anterior thoracotomy/minithoracotomy, TCRAT, total/complete coronary revascularization). Eligible reports were clinical series describing multivessel CABG performed through a left anterior thoracotomy within the TCRAT concept and reporting early clinical outcomes; we excluded single-vessel MIDCAB-only reports, purely off-pump series, isolated case reports, and manuscripts with insufficient outcome reporting. Potentially overlapping cohorts were assessed, and duplicate data were excluded. Methodological quality was appraised at the study level; comparative observational studies were evaluated using the Newcastle–Ottawa Scale, whereas single-arm case series were judged against standard case-series quality domains (selection, outcome definition/adjudication, and completeness of reporting), and these considerations informed the strength of our narrative conclusions.

To date, seven surgical groups have published clinical outcomes of TCRAT [15,16,17,18,19]. For the present analysis, duplicate data from overlapping series were excluded (Table 1). Collectively, these studies encompass 2282 patients treated within a relatively short time frame, averaging approximately 100 cases per center per year. To provide an integrated overview, key perioperative and outcome parameters from all available series were summarized using descriptive statistics, and weighted means were calculated to account for differences in cohort sizes across studies. Such numbers suggest that TCRAT is evolving into a routinely performed coronary revascularization strategy. Indeed, all institutions that have established TCRAT programs report it as their preferred standard approach for multivessel CABG [12,13,14,15,16,17,18].

Baseline characteristics across series are largely comparable and representative of contemporary, unselected, elective CABG populations. Common exclusion criteria include a calcified aorta, redo procedures, and emergencies. Notably, several reports include patients with severe obesity, unfavorable chest anatomy, or impaired ventricular function—groups typically excluded from minimally invasive beating-heart CABG.

Intraoperatively, TCRAT is associated with longer operative, CPB, and cross-clamp times than conventional CABG. However, this does not translate into prolonged intensive care unit (ICU) or hospital stays. The approach allows individualized graft configurations, with LITA use ranging from 94.8% to 100%. Although RITA utilization remains rare, the radial artery is frequently employed [15,17,18], reaching 65.7% in one series and yielding a high rate of multiarterial grafting [15]. Both conventional aortic side-clamp and composite T- or Y-graft techniques are reported. The mean number of distal anastomoses ranges between 2.5 and 3.4, with up to five achieved in some series [15,16,17,18], consistent with the completeness of revascularization seen in standard on-pump sternotomy CABG [21].

The 30-day mortality is 0.7% (range, 0–1.2%), comparing favorably with outcomes from contemporary conventional CABG [21]. The overall stroke rate of 0.7% is likewise remarkably low for on-pump procedures, likely reflecting patient selection and the routine use of preoperative CT-guided perfusion strategies, including frequent axillary cannulation and the avoidance of high-risk aortic manipulation.

Beyond neurological safety, ongoing investigations have explored organ-specific outcomes. Pulmonary analyses revealed a higher incidence of pleural effusion in TCRAT patients, though without adverse clinical impact [22]. Similarly, renal outcome studies found no increase in postoperative dysfunction despite longer operative and CPB durations [23]. ICU and total hospital stays were shorter compared to conventional sternotomy CABG [16,19,23]. These findings collectively support TCRAT as a reproducible, safe, and physiologically advantageous sternum-sparing approach to complete multivessel coronary revascularization.

Mid-term outcomes of TCRAT have been reported by two surgical groups. In the first series [24], complete follow-up was achieved in 392 patients with a mean duration of 15.2 ± 10.7 months (range, 0.1–39.5), showing an overall rate of major adverse cardiac and cerebrovascular events (MACCE) of 8.7%. In the second study [18], late mortality occurred in 4 patients (2.7%), two of whom died from non-cardiac causes (malignancy and pulmonary embolism) and one from cardiac arrest. Nine patients (6%) required late coronary reintervention, all successfully treated with percutaneous coronary intervention, and no postoperative strokes were reported. Kaplan–Meier analysis demonstrated 1-year and 5-year survival rates of 99.1% and 97.5%, respectively, with freedom from MACCE of 97.3% and 95.0%. Collectively, these results confirm excellent mid-term survival and event-free outcomes after TCRAT, consistent with one-year results from landmark myocardial revascularization trials [25,26] and comparable to contemporary multicenter CABG registries [21]. Nevertheless, continued long-term surveillance remains essential to further define the durability and prognostic implications of the procedure.

The principal advantage of the TCRAT technique over conventional CABG lies in the complete avoidance of median sternotomy. Consequently, the risks of superficial or deep sternal wound infection and postoperative sternal instability [27] are entirely eliminated. The absence of a sternotomy also facilitates faster recovery, earlier return to physical activity, and greater overall acceptance of surgical revascularization among both patients and physicians [14]. Future investigations should further evaluate whether specific subgroups (women, obese patients, those with reduced ejection fraction, advanced age, or those with chronic obstructive pulmonary disease) derive particular benefit from this sternum-sparing strategy. Moreover, avoiding sternotomy-related complications could substantially reduce the significant healthcare costs associated with sternal wound infection and its sequelae [28,29].

## 4. TCRAT—Combination with Valve Procedures: Concepts and Perspectives

As an on-pump, arrested-heart procedure, TCRAT inherently permits combination of minimally invasive multivessel CABG with a variety of other cardiac operations [30]. Several configurations have been described to accommodate concomitant interventions, depending on the target pathology and institutional experience. Techniques employing a partial upper sternotomy for central arterial cannulation, aortic cross-clamping, and proximal anastomoses, combined with a left anterior minithoracotomy for distal coronary grafting, have enabled simultaneous aortic valve replacement and CABG without the need for full sternotomy [31]. This hybrid configuration maintains conventional central perfusion while minimizing surgical trauma.

To achieve a fully sternum-sparing concept, bilateral minithoracotomy strategies have been developed, performing TCRAT via the left incision and valve procedures through a right-sided access [30]. These approaches allow independent exposure of the coronary and valvular fields while preserving the advantages of limited access and cosmetic benefit.

Recent advances have further expanded the scope of minimally invasive combined operations. Several groups have demonstrated the feasibility of performing complete coronary revascularization together with aortic or mitral valve surgery through a single left anterior minithoracotomy [32,33,34]. In these procedures, the arrested and decompressed heart provides excellent exposure for both coronary and valvular structures. Use of sutureless or rapid-deployment aortic prostheses significantly simplifies implantation within the restricted operative field, compensating for limited visualization and reducing cross-clamp time. Similarly, minimally invasive mitral valve repair or replacement can be achieved through the same incision by exploiting the direct transatrial route under full cardioplegic arrest, allowing complete revascularization and valve correction in a single setting.

Looking ahead, integration of robotic and endoscopic assistance represents the next evolutionary step in combined TCRAT procedures. Robotic harvesting of internal thoracic arteries and robotic or endoscopic valve exposure may further optimize visualization, ergonomics, and precision within the constrained thoracic space, potentially reducing operative time and expanding the applicability of this approach to a wider patient population.

Collectively, these developments underscore the potential of TCRAT as a platform for multicomponent minimally invasive cardiac surgery, enabling safe, complete, and physiologic revascularization together with complex valve interventions, while entirely avoiding median sternotomy.

## 5. Summary

TCRAT represents the most recent advancement in sternum-sparing, minimally invasive CABG performed under CPB with cardioplegic arrest. This technique enables complete anatomical coronary revascularization and facilitates multiarterial grafting through a left anterior minithoracotomy, even in unselected patients with multivessel coronary disease. Early and mid-term outcomes have been highly favorable, while long-term results are awaited to confirm durability. Future refinements are expected to include combined procedures and integration of robotic or endoscopic assistance. With its reproducibility, safety profile, and applicability, the TCRAT approach holds strong potential to become a standardized routine technique, marking a significant step forward in the evolution of sternum-sparing cardiac surgery.

## Figures and Tables

**Table 1:** Comparison of published clinical series of Total Coronary Revascularization via left Anterior Thoracotomy.

	Babliak et al. *	Demianenko et al. [15]	Demirsoy et al. [17]	Çaynak et al. **	Arslanhan et al. [18]	Demirkiran et al. [19]	Sicim et al. [16]	All Series (Weighted Mean)
**Patients, n**	800	502	230	412	98	108	132	2282
**Inclusion period**	7/2017–6/2025	11/2019–5/2024	7/2020–9/2022	7/2019–12/2023	4/2022–4/2024	2/2021–9/2022	4/2018–2/2022	2017–2025
**Age, years**	62.9 ± 9.3	67.2 ± 9.6	60.2 ± 10.3	56.4 ± 9.7	61.7 ± 9.6	57.1 ± 8.8	58.5 ± 8.7	61.8 ± 9.4
**Male, n (%)**	664 (83.0)	436 (86.9)	206 (89.5)	146 (79.3)	122 (81.3)	95 (88.0)	95 (74.2)	1966 (86)
**BMI;**	30.0 ± 4.6	28.4 ± 4.8	28.7 ± 4.4	NA	29.0 ± 5.9	NA	25.3 ± 5.4	28.4 ± 5.0
**LVEF (%)**	51.3 ± 9.3	49.0 ± 9.8	53.4 ± 9.6	NA	NA	55.9 ± 9.1	NA	51 ± 9
**Intraoperative characteristics**								
**Dist. anastomoses/patient**	3.0 ± 0.7	3.0 ± 0.8	3.0 ± 0.9	3.3 ± 0.6	2.3 ± 1.2	2.5 ± 1.0;	3.4 ± 0.6	3.0 ± 0.8
**LITA, n (%)**	774 (96.7)	494 (98.4)	225 (97.8)	406 (98.5)	98 (100)	104 (96.3)	132 (100)	2233 (97.9)
**RITA, n (%)**	11 (1.4)	0	2 (0.9)	0	0	0	0	13 (0.6)
**Radial artery, n (%)**	237 (29.6)	330 (65.7)	88 (38.2)	0	19 (19.4)	0	0	672 (29.5)
**Cross-clamp time, min**	73.5 ± 20	95.8 ± 32.7	78 ± 22	76.6 ± 17.5	69.5 ± 20.3	77 ± 38	86 ± 13	80 ± 25
**CPB time, min**	151.9 ± 35.3	155.7 ± 42.2	151 ± 45	140 ± 23	152 ± 43.5	168 ± 69	152 ± 36	155 ± 40
**Total op. time, min**	278.5 ± 52.3	322.9 ± 75	258 ± 60	NA	NA	NA	NA	290 ± 65
**Postoperative characteristics**								
**ICU stay, days**	1.8 ± 1.0	2.3 ± 4.6	1.5 ± 1.3	1.3 ± 0.5	0.8 ± 0.3	1.7 ± 1.8	1.1 ± 0.4	1.8 ± 1.9
**Total hospital stay, days**	6 ± 1.9	NA	5.5 ± 2.2	5.4 ± 1.4	6.9 ± 2.4	6.8 ± 4.2	5.2 ± 2.1	5.8 ± 2.3
**Rethoracotomy, n (%)**	9 (1.1)	35 (7.0)	5 (2.2)	NA	1 (1.0)	7 (6.4)	6 (4.5)	63 (3.4)
**Stroke, n (%)**	3 (0.4)	3 (0.6)	3 (1.3)	NA	0	2 (1.8)	2 (1.5)	13 (0.7)
**30-day mortality, n (%)**	3 (0.4)	6 (1.2)	2 (0.9)	4 (1.0)	0	0	1 (0.7)	16 (0.7)
**Acute renal failure, n (%)**	4 (0.5)	7 (1.4)	NA	NA	0	NA	6 (4.5)	17 (1.0)
**Wound infection, n (%)**	NA	6 (1.2)	4 (1.8)	NA	0	3 (2.7)	2 (1.5)	15 (1.4)

BMI—body mass index, LVEF—left ventricle ejection fraction, LITA—left internal thoracic artery, RITA—right internal thoracic artery, CPB—cardiopulmonary bypass, ICU—intensive care unit, *—Data first reported at the ISMICS 2025 Annual Meeting (oral presentation), **—Data presented as a poster at the ISMICS 2024 Annual Meeting.

## Data Availability

No new data were created or analyzed in this study. Data sharing is not applicable to this article.

## Supplementary Materials

The following supporting information can be downloaded at: https://www.mdpi.com/article/10.3390/jcdd13010028/s1, Video S1: Left Anterior Minithoracotomy and ITA Harvesting, Video S2: Peripheral Cannulation, Cardiopulmonary Bypass, and Transthoracic Aortic Cross-Clamping, Video S3: Exposure Maneuvers.

## Abbreviations

CABG	coronary artery bypass grafting
CPB	cardiopulmonary bypass
ICU	intensive care unit
IVC	inferior vena cava
LITA	left internal thoracic artery
LPV	left pulmonary veins
MACCE	major adverse cardiac and cerebrovascular events
MIDCAB	minimally invasive direct coronary artery bypass
RITA	right internal thoracic artery
TCRAT	Total Coronary Revascularization via left Anterior Thoracotomy
